# Advancements in Cancer Care by Exploring Multimodality Imaging Techniques and their Applications

**DOI:** 10.2174/0115734056384570250515050115

**Published:** 2025-06-23

**Authors:** Ramesh Kumar, Ashish Kumar Singh, Manish Kumar Singla, Anupma Gupta, El-Sayed M. El-kenawy, Amal H. Alharbi

**Affiliations:** 1 Department of Interdisciplinary Courses of Engineering, Chitkara University Institute of Engineering & Technology, Chitkara University, Rajpura, Punjab, India; 2 Jadara University Research Center, Jadara University, Irbid, Jordan; 3 Departments of Electronics and Communication Engineering, Graphic Era (Deemed to be University), Dehradun, Uttarakhand-248001, India; 4 Department of Biosciences, Saveetha School of Engineering, Saveetha Institute of Medical and Technical Sciences, Chennai, India; 5 Applied Science Research Center, Applied Science Private University, Amman, Jordan; 6 School of ICT, Faculty of Engineering, Design and Information & Communications Technology (EDICT), Bahrain Polytechnic, Isa Town, Bahrain; 7 Department of Computer Sciences, College of Computer and Information Sciences, Princess Nourah bint Abdulrahman University, Riyadh, Saudi Arabia

**Keywords:** Advanced Cancer Care, Image Processing, Diagnosis, Therapeutics, Cancer Imaging, Cancer Treatments, Clinical Application, AI-driven

## Abstract

Advancements in multimodality imaging have significantly improved cancer diagnosis, treatment planning, and patient management. This review explores the integration of imaging techniques, such as MRI, CT, and PET, alongside emerging technologies like radiomics and AI to provide comprehensive insights into tumor characteristics. By combining imaging data with laboratory tests, clinicians can achieve more accurate cancer staging and personalized treatment strategies. Noninvasive image-guided therapies and early detection through screening programs have shown promise in reducing mortality and treatment-related side effects. This review highlights the importance of collaboration between academia, biotechnology, and the pharmaceutical industry to drive innovation in cancer imaging. Future advancements in imaging technologies, combined with interdisciplinary collaborations, hold promise for further improving cancer diagnosis, treatment, and patient outcomes, with AI-driven tools further enhancing precision oncology and patient care.

## 1 INTRODUCTION

**Fig. (1):**
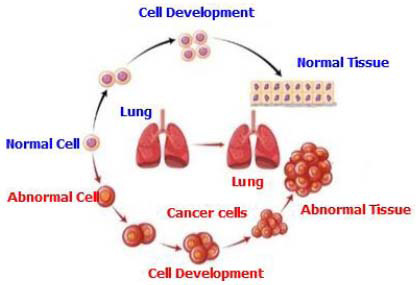
Cell development (normal *vs*. abnormal).

Cancer is a diverse and intricate disease driven by the unchecked growth and migration of abnormal cells (Fig. **1**). These cellular abnormalities originate from genetic mutations that interfere with the stringent control mechanisms governing cell division and differentiation [1]. As these mutations accumulate, cells lose their ability to regulate proliferation and adhesion, resulting in the development of tumors and the potential for metastasis, the spread of cancerous cells to distant locations throughout the body [2]. Cancer can develop in any organ or tissue, leading to a variety of symptoms depending on the affected region and the stage of the disease.

Cancer exerts a substantial and growing global impact. In 2020, an estimated 19.3 million new cancer cases were diagnosed worldwide, with the disease accounting for over 10 million deaths [3-5]. This means that globally, approximately one in seven deaths is due to cancer, making it the second leading cause of mortality after cardiovascular diseases [4]. The global burden of cancer is expected to rise further in the coming decades, driven by factors, such as population aging, rapid urbanization, and increasing prevalence of modifiable risk factors, including unhealthy lifestyle choices. These trends underscore the urgent need for intensified global collaboration in cancer research, prevention, and early detection strategies to combat this significant public health challenge.

Cancer imaging plays a pivotal role across all stages of oncological care, profoundly influencing clinical decision-making and patient prognosis. Modern imaging techniques offer comprehensive insights, encompassing anatomical localization, physiological dynamics, and molecular profiles of neoplastic tissues [5]. At the diagnostic stage, these imaging modalities are vital for early cancer detection. Population-based screening programs, such as mammography for breast cancer and low-dose CT for lung cancer, enable the identification of asymptomatic lesions, facilitating intervention at stages when treatment is most effective and survival rates are highest [6, 7]. Once a tumor is identified, advanced imaging techniques provide precise histopathological correlation and inform risk stratification. Magnetic Resonance Imaging (MRI) delivers exceptional soft-tissue contrast for differentiating benign from malignant lesions, while Fluorodeoxyglu-cose–Positron Emission Tomography (FDG-PET) measures metabolic activity, offering valuable insights into tumor grade and therapeutic response [8].

Despite advancements in treatment, early detection and accurate diagnosis remain significant challenges in cancer care. Conventional imaging techniques, such as X-rays and CT scans, often lack the sensitivity and specificity required for precise tumor characterization, especially in complex cases [6, 7]. This shortcoming highlights a critical gap in current diagnostic strategies, where multimodality imaging emerges as a transformative approach. Imaging is indispensable in oncology, providing essential insights into tumor biology, anatomical structures, and functional dynamics. For example, mammography has been shown to reduce breast cancer mortality by 20–30% in screened populations (American Cancer Society), underscoring the life-saving impact of early detection. However, the limitations of single-modality imaging, such as the poor soft-tissue contrast of CT scans or the limited depth penetration of ultrasound, emphasize the need for integrated imaging solutions. Multimodality imaging, which combines complementary techniques like MRI, PET, and novel technologies, offers a promising pathway to address these gaps and improve patient outcomes [9].

This review addresses a critical gap in the existing literature by providing a comprehensive analysis of both established and emerging imaging modalities, such as radiomics, multimodality imaging, and hybrid systems like PET/MRI. Unlike previous reviews that focus on individual techniques, this work emphasizes the integration of multiple imaging approaches to achieve a holistic understanding of cancer biology and treatment response. These innovations hold promise for improving patient outcomes and bridging the gap between research and clinical practice. Key points include the global cancer burden of 19.3 million new cases in 2020 [1], the limitations of traditional imaging in complex cases, and the role of multimodality imaging in overcoming these challenges. By focusing on integration and emerging technologies, this review aims to advance the field of oncologic imaging and address gaps such as over-reliance on single-modality imaging and limited discussion of AI and hybrid systems [10, 11].

This paper provides a comprehensive evaluation of clinical imaging modalities within the realm of cancer diagnosis and therapeutic management, emphasizing recent advancements and emerging trends. While foundational principles and workflows are included for context, the primary focus is on the evolution of imaging technologies and their increasing sophistication and impact on clinical outcomes. This evaluation is crucial for tracking the progression of imaging technology, identifying current platform deficiencies, and guiding the development of next-generation solutions in cancer care. The paper highlights innovative imaging approaches, including hybrid modalities, such as PET/MRI and PET/CT, and their accompanying software systems, which significantly enhance diagnostic precision and treatment monitoring. Additionally, the manuscript examines the integration of advanced imaging biomarkers with Artificial Intelligence (AI) algorithms, facilitating early detection, tumor characterization, and individualized treatment planning. Techniques like functional MRI (fMRI), radiomics, and theranostic imaging are also explored for their transformative potential in predictive and adaptive cancer treatment strategies. By underscoring these contemporary advancements, the paper offers a forward-looking perspective on the pivotal role of imaging in advancing both oncology research and clinical practice.

## 2 MULTIMODALITY IMAGING IN CANCER CARE: A LITERATURE REVIEW

Cancer remains a major global health burden, underscoring the need for continual advances in diagnosis, treatment, and management strategies. In this landscape, multimodality imaging has emerged as a transformative tool in cancer care. This comprehensive review critically examines the current state of the literature on the integration of various imaging techniques in oncology [7-10]. To ensure a systematic and evidence-based approach, the literature review was conducted using a rigorous methodology. Studies published between 2015 and 2023 were prioritized, focusing on cutting-edge developments in multimodality imaging systems and their clinical applications in oncology [9-12]. Key search terms included “multimodality imaging in cancer,” “radiomics,” “multimodality imaging,” “PET/MRI,” and “theranostic.” Inclusion criteria included peer-reviewed articles, clinical trials, and reviews discussing the integration of multiple imaging modalities for cancer diagnosis, treatment planning, and monitoring (Table **1**). Studies unrelated to clinical applications or solely addressing single-modality imaging were excluded. From an initial pool of 120 articles, 80 were selected for in-depth analysis based on their relevance and contribution to the field. This rigorous selection process ensured that the review provided a comprehensive and evidence-based perspective, free from unverified narrative accounts.

A growing body of research highlights the significant impact of multimodality imaging on early cancer detection. Studies have demonstrated the effectiveness of combining modalities, such as low-dose CT scans and fluoroscopy, for lung cancer screening [9]. Similarly, the combination of mammography with ultrasound or MRI has shown promise in improving breast cancer detection, particularly in women with dense breast tissue [10]. These findings underscore the potential of multimodality imaging to identify potentially malignant lesions at earlier stages, leading to improved patient outcomes [11]. Table **1** below summarizes the potential benefits of multimodality imaging across various aspects of cancer care.

## 3 METHODOLOGY FOR CANCER IMAGING MODALITIES

**Fig. (2):**
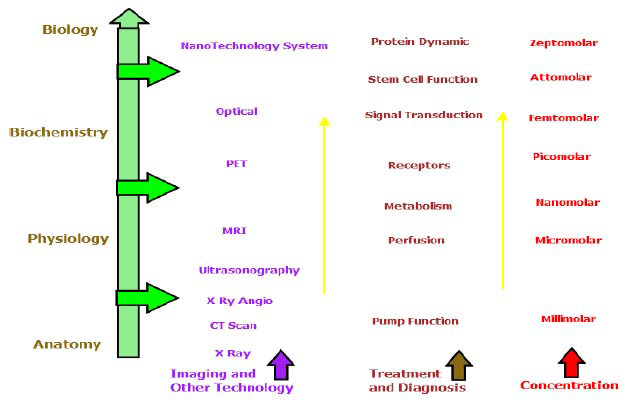
Cancer diagnosis according to imaging modalities, such as all multimodalities imaging.

The foundation of oncologic diagnosis and clinical management relies on a well-established array of imaging modalities. Conventional techniques, including radiography (X-ray), Computed Tomography (CT), Magnetic Resonance Imaging (MRI), ultrasonography, and nuclear medicine imaging, provide essential diagnostic information throughout the cancer care continuum [43-46]. These modalities can be systematically categorized into three classes: (1) conventional radiographic techniques, (2) nuclear medicine imaging, and (3) non-ionizing electromagnetic-based modalities (Fig. **2**).

Each imaging modality plays a distinct and essential role in clinical oncology, contributing to lesion detection, tumor staging, treatment response evaluation, and image-guided interventions, collectively enhancing diagnostic precision and therapeutic outcomes [43, 44]. However, these modalities differ significantly in terms of radiation exposure and associated risks. Low-dose modalities, such as ultrasound and MRI, do not employ ionizing radiation, offering safer imaging options, especially for vulnerable populations like children and pregnant patients, though they have certain limitations in application. Moderate-dose techniques, such as CT scans, deliver doses of approximately 2–20 mSv per scan. While generally safe, these modalities pose a small but notable risk, particularly for pediatric patients and those undergoing repeated imaging procedures. High-dose modalities, including PET and SPECT scans, utilize radiotracers and can expose patients to doses of up to 25 mSv per scan. Such higher doses increase the potential for long-term cancer risk, especially in populations with heightened radiosensitivity. Although immediate radiation-induced complications are rare, cumulative exposure over time can lead to a dose-dependent increase in oncogenic potential, with pediatric patients being particularly vulnerable.

This review offers a systematic evaluation of Artificial Intelligence (AI) algorithms, focusing specifically on their clinical applications in oncological image interpretation and radiotherapy planning. These algorithms, built on machine learning and deep learning frameworks, are designed to enhance diagnostic accuracy, automate complex image analysis tasks, and enable personalized treatment strategies. Convolu-tional Neural Networks (CNNs) and transformer-based models, in particular, have shown significant promise in tumor detec-tion, segmentation, and classification, while predictive models assist in assessing treatment response and disease progression.

Despite these advancements, several challenges continue to impede the widespread clinical adoption of AI. Variability in imaging protocols and data heterogeneity, along with the limited availability of well-annotated datasets, introduce biases that hinder the generalizability of AI models. Furthermore, the “black box” nature of many deep learning algorithms raises concerns about interpretability and clinical confidence, emphasizing the need for explainable AI techniques to ensure transparency in decision-making. Regulatory and ethical considerations, including patient data privacy and the validation of AI models across diverse populations, add additional complexity to integrating AI into routine oncology practice. By examining both the transformative potential and the inherent limitations of AI in multimodality cancer imaging, this paper provides a balanced perspective on the critical challenges that must be overcome to translate technological innovation into real-world clinical impact.

### 3.1 Conventional Imaging Techniques

**Fig. (3):**
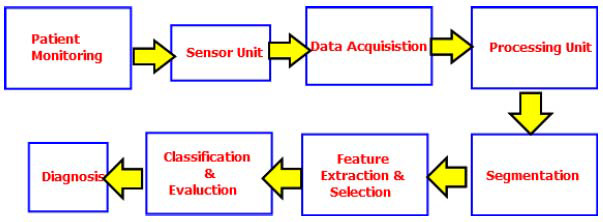
Conventional imaging techniques.

Conventional imaging modalities represent a cornerstone of cancer diagnosis and management, as illustrated in the conceptual framework of imaging applications (Fig. **3**). These established techniques—X-ray, Computed Tomography (CT), Magnetic Resonance Imaging (MRI), and ultrasound—provide clinicians with critical anatomical and physiological information throughout the disease continuum [5]. X-rays offer a rapid and cost-effective means of initial screening, while CT scans deliver detailed cross-sectional images that are essential for accurate tumour staging and treatment planning. MRI is particularly effective for visualizing soft tissues, making it indispensable for assessing the involvement of the brain or spinal cord. Additionally, ultrasound, a safe and readily accessible modality, plays a vital role in breast cancer screening and image-guided biopsies [47, 48].

#### 3.1.1 X-ray Imaging Technique

Conventional X-ray imaging has long served as a cornerstone in cancer diagnosis, with over a century of use [49]. This simple and cost-effective technique relies on ionising radiation to image both bone and soft tissue structures. While X-rays lack high specificity, they can reveal suspicious lesions in the lungs, bones, and other areas, prompting further investigations with more advanced imaging modalities. X-ray guidance also remains indispensable for interventional procedures such as tumour biopsies, where real-time needle tracking is essential. Despite the emergence of high-resolution imaging techniques, traditional X-rays continue to be widely used as an initial screening tool in cancer care due to their speed, accessibility, and cost-effectiveness.

When X-rays pass through the body, they interact differently with various tissues depending on their composition and density. Regions with higher electron density, such as bones and metal implants, produce stronger X-ray scattering signals compared to softer tissues. This scattering reduces the number of X-ray photons that reach the detector film, making these denser structures appear brighter on X-ray images [51], as illustrated in Fig. (**4**). However, this density-dependent contrast can create challenges when distinguishing soft tissues that have similar electron densities—such as tissues surrounding blood vessels—since they appear similar on conventional X-rays.

To address this limitation, contrast agents are often used during X-ray imaging. These agents are typically dense liquids containing elements with high atomic numbers, such as iodine or barium. The “heavy atoms” in these contrast agents interact strongly with X-rays, increasing the contrast between blood vessels and the surrounding soft tissues [50]. This enhanced contrast helps physicians visualize vascular structures and other subtle anatomical features more clearly.

X-rays offer several advantages and limitations in cancer detection. Their affordability, speed, and widespread availability make them a valuable first-line screening tool. For instance, chest X-rays are commonly used to detect suspicious lung nodules that may require further evaluation [48]. Additionally, fluoroscopy, which employs real-time X-ray imaging, enhances the accuracy of biopsies and minimally invasive procedures, improving patient outcomes [49]. However, X-rays lack the specificity and detailed resolution of advanced imaging techniques, such as CT scans, making it difficult to reliably differentiate between benign and malignant tumors [50]. Moreover, because X-rays involve ionizing radiation, repeated exposure necessitates a careful risk-benefit assessment to minimize potential long-term health risks while maximizing diagnostic value [48-50].

#### 3.1.2 Ultrasound Imaging Technique

**Fig. (5):**
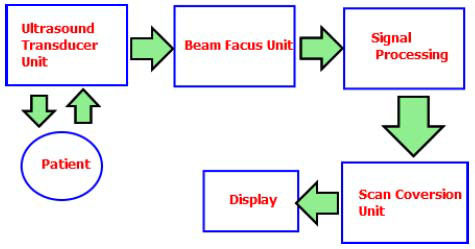
Working layout of the USG imaging techniques.

Ultrasound imaging uses high-frequency sound waves (typically 2–18 MHz) that travel through tissues, with the reflected echoes captured and processed to create real-time anatomical images. This technology is especially effective in distinguishing between solid structures and fluid-filled areas. As a non-invasive and radiation-free modality, ultrasound safely visualizes tissue morphology, organ movement, and blood flow (Fig. **5**). It serves as a valuable adjunct in clinical decision-making across a wide range of medical conditions [26]. In cancer care, ultrasound is a versatile and patient-friendly tool suitable for multiple stages of management. Unlike ionizing radiation-based techniques, such as X-rays, ultrasound offers a safe option for repeated monitoring throughout treatment [16].

Ultrasound excels in real-time visualization of soft tissues, making it invaluable for detecting potentially malignant masses or abnormalities that may be missed by other imaging methods. A prime example is breast cancer screening, where ultrasound effectively identifies suspicious lumps. Early detection through ultrasound facilitates timely biopsies, potentially improving patient outcomes. Beyond detection, its real-time imaging capability makes ultrasound ideal for guiding biopsies. Physicians can accurately direct needles to targeted lesions, ensuring precise tissue sampling for definitive diagnosis. This accuracy is crucial for distinguishing between benign and malignant tumors and for informing treatment decisions. Ultrasound-guided biopsies are commonly used for breast lesions, prostate abnormalities, and various other organs accessible by ultrasound waves [26].

While ultrasound may not provide the detailed resolution of CT scans or MRIs, it remains a valuable tool for assessing tumor characteristics important for cancer staging. By visualizing tumor size, location, and its relationship to surrounding structures, such as blood vessels or lymph nodes, ultrasound helps determine the extent of cancer spread [18]. This information is critical for standardized cancer staging. For instance, ultrasound is frequently used to evaluate lymph node involvement in different cancers, assisting in selecting the most appropriate treatment strategy [45].

It is vital to recognize the limitations of ultrasound. Its effectiveness can be reduced by factors such as body fat or overlying bone. Ultrasound waves cannot penetrate deeply into the body, making them less suitable for visualizing deeply situated organs. Additionally, ultrasound images may not provide the same level of detail as CT scans or MRIs, particularly for complex anatomical structures. Nevertheless, ultrasound is a versatile tool in many stages of cancer care. Its safety, affordability, and real-time visualization capabilities make it invaluable for early detection, image-guided procedures, and tumor staging. Although it has its limitations, ultrasound remains a cornerstone of cancer imaging, often used in conjunction with other modalities for a comprehensive diagnostic approach [26].

#### 3.1.3 Computed Tomography (CT) Imaging Technique

**Fig. (6):**
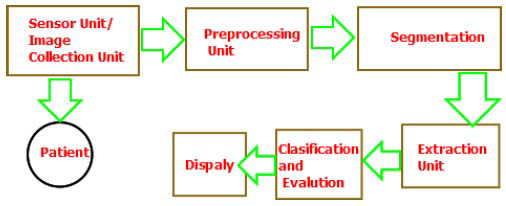
Block diagram of commuter tomography imaging techniques.

Computed Tomography (CT) scans generate detailed cross-sectional images of the body. The term “tomography” originates from the Greek word meaning “slice.” During a CT scan, a narrow X-ray beam rotates around the patient’s body while detectors measure the intensity of X-rays passing through different tissues [52]. A computer then processes this data to construct a detailed “slice” image. Developed in the mid-1970s, CT technology revolutionized medical imaging by providing precise anatomical information, clearly depicting air spaces, bones, and soft tissues. To create three-dimensional images, the X-ray source continuously rotates around the patient as detectors capture X-rays from multiple angles. Unlike traditional X-rays that rely on film, CT scans use digital detectors (Fig. **6**) that convert X-ray energy into electrical signals. This digital process eliminates the need for film development and offers enhanced precision and flexibility for image manipulation [24-28].

CT has revolutionized cancer imaging by providing exceptional detail and versatility. Unlike traditional X-rays, which produce a flat image, CT scans generate detailed cross-sectional views of the body using X-rays. The resulting images allow physicians to visualize internal structures in three dimensions, making CT an essential tool for cancer diagnosis, staging, treatment planning, and monitoring. A closer look at the importance of CT in cancer imaging, as well as the crucial role of evaluating high-quality CT scans, is given below [26-30].

CT scans provide high-quality anatomical detail, allowing for the visualization of tumors, including their size, shape, and location within the body. This information is crucial for early detection, as CT scans can reveal small tumors that might be missed by other imaging modalities like X-rays. Early detection enables prompt intervention and potentially improves patient outcomes [27]. In tumor characterization, CT scans can sometimes distinguish between benign and malignant tumors based on factors, such as size, density, and blood flow patterns [28]. This helps reduce the need for invasive biopsies or guides specific treatment approaches. CT scans also play a vital role in cancer staging, a standardized system used to determine the extent of cancer spread. By visualizing the involvement of lymph nodes and other organs, CT scans help classify the cancer stage, which informs treatment decisions [53, 54].

While standard CT scans provide valuable anatomical information, contrast-enhanced CT scans offer even greater insights. During a contrast-enhanced CT scan, a contrast agent, usually an iodine-based dye, is injected intravenously. This contrast agent absorbs X-rays differently than surrounding tissues, making blood vessels and certain abnormalities seem brighter on the CT scan pictures [52]. This enhanced comparison permits improved tumour delineation.

##### 3.1.3.1 Improved Tumor Delineation

Enhanced CT scans can clearly outline the margins of a tumor, distinguishing it from surrounding healthy tissues [55]. This is especially useful for tumors located near critical structures. In vascular evaluation, the contrast agent flowing through blood vessels enables visualization of blood flow patterns within and around the tumor. This information is vital for treatment planning, particularly for therapies that target tumor blood supply [23-28]. Additionally, contrast-enhanced CT scans excel at detecting tumors that have spread (metastasized) to other organs, such as the liver, lungs, or lymph nodes. Early detection of metastases is crucial for determining the best treatment strategy [52, 53].

Despite their advantages, CT scans do have limitations. They involve exposure to ionizing radiation, which carries a small risk of inducing cancer, especially with repeated scans. Additionally, CT scans may not be suitable for all patients due to allergies to contrast agents or certain medical conditions. However, CT scans, particularly contrast-enhanced CT scans, have become fundamental tools in cancer imaging. Their ability to provide detailed anatomical information, differentiate tissue types, and assess blood flow patterns helps doctors make accurate diagnoses, plan treatments, and monitor patients throughout their cancer care.

#### 3.1.4 Magnetic Resonance Imaging (MRI) Technique

Similar to CT scans, MRI produces cross-sectional images but without the associated radiation exposure. One of the primary reasons for the high cost of MRI scans is the need for a powerful superconducting magnet. This magnet generates a strong magnetic field, which aligns the protons within the body’s atoms. Once aligned, radio waves are pulsed through the targeted area [52]. The protons respond to these radio waves by emitting faint signals, which are then detected and converted into a digital image by the system’s computer, as illustrated in Fig. (**7**).

MRI has become a powerful tool in the fight against cancer. Unlike X-rays, which use ionizing radiation, MRI provides a safe and non-invasive method to visualize internal structures with exceptional detail. This precise visualization makes MRI invaluable for cancer evaluation, offering several key advantages. It delivers superior soft tissue contrast by exploiting the magnetic properties of tissues and enables multiplanar imaging (axial, coronal, and sagittal planes), which is crucial for thorough tumor assessment. Additionally, advanced functional techniques, such as Diffusion-Weighted Imaging (DWI) and perfusion MRI, provide important physiological insights into tumor characteristics.

MRI is particularly effective for detecting and characterizing tumors in soft tissue organs, including the brain, liver, muscles, and breasts [23-40]. For example, MRI can differentiate between benign and malignant breast lesions based on their distinct signal characteristics [38]. The ability to generate images from multiple planes allows physicians to view tumors from various angles, improving their understanding of tumor size, precise location, and relationship to surrounding structures [3]. This multiplanar capability is especially valuable in complex anatomical regions and surgical planning. Functional MRI techniques like DWI further enhance tumor evaluation by assessing tumor cellularity and blood flow. These insights assist in distinguishing benign from malignant tumors and inform treatment strategies, particularly those targeting tumor blood supply [40].

A key limitation of MRI is its high cost, which requires careful consideration. MRI machines are expensive to purchase, maintain, and operate, leading to higher examination fees compared to other imaging modalities like CT scans. Additionally, MRI scans generally take longer to perform, which can be uncomfortable for patients experiencing pain or anxiety. The enclosed space within the MRI scanner may also cause claustrophobia in some individuals [54]. MRI also has certain imaging limitations. It is less effective at visualizing tissues, such as bones and air-filled structures like the lungs, due to their physical properties [55]. Safety concerns must also be considered, as the strong magnetic fields produced by MRI systems can pose risks to patients with specific medical implants or metallic devices. Despite these challenges, MRI remains an invaluable tool in cancer evaluation, offering superior soft tissue contrast, multiplanar imaging, and functional capabilities. To overcome its limitations, MRI is often used alongside other imaging modalities, such as CT scans and ultrasound, to provide a more comprehensive assessment of cancer care [23-40].

### 3.2 Nuclear Medicine Techniques

#### 3.2.1 Positron Emission Tomography

Positron Emission Tomography (PET) is a nuclear medicine imaging technique that produces three-dimensional images reflecting functional processes within the body. As a branch of nuclear medicine, PET employs trace amounts of radioactive tracers to diagnose and manage a variety of conditions, including many cancer types, cardiovascular diseases, and other physiological disorders [52]. This modality enables non-invasive, cross-sectional imaging that localizes and quantifies metabolic activity.

In PET imaging, a radioisotope injected into the body emits positrons, which collide with nearby electrons, resulting in mutual annihilation. This process produces two gamma rays of equal energy that travel in opposite directions. PET scanners are equipped with detectors arranged around the patient to simultaneously detect these gamma rays. Using reconstruction algorithms similar to those in CT imaging, the system generates detailed functional images, as illustrated in Fig. (**8**) [55].

PET scans utilize specialized imaging technology to detect cellular metabolic activity. The scanner features a large, donut-shaped gantry with a central opening, allowing patients to lie comfortably while the detector rotates around them. The procedure involves injecting a safe radioactive tracer, typically a glucose analog, which accumulates in target tissues or organs based on their metabolic activity. As these tracers decay, they emit positrons that the scanner detects, creating detailed 3D images of biological processes. Once inside the body, the tracers break down, emitting tiny debris called positrons. As positrons engage with close-by electrons, they release gamma rays. The PET scanner detects those gamma rays and makes use of them to create an in-depth 3-D photograph of the targeted place. The quantity of tracer taken up by tissues determines how vivid they appear in the image. PET scans can make use of numerous tracers relying on the physician's recognition. For example, tracers mimicking oxygen glide might be used for blood studies, at the same time as those mimicking glucose might be used for cancer detection (for the reason that cancer cells tend to be more energetic and consume extra glucose). Conversely, some diseases can lead to decreased tracer uptake due to decreased cellular function [23-30].

Cancer diagnosis and treatment heavily depend on accurate imaging techniques, with Positron Emission Tomography–Computed Tomography (PET/CT) emerging as a vital tool in oncology. PET/CT combines two complementary modalities: PET, which detects gamma rays emitted by a safe radioactive tracer, often a glucose analog, injected into the bloodstream, and CT, which uses X-rays to produce detailed cross-sectional images of internal structures. Since cancer cells exhibit higher metabolic activity, they absorb more of the tracer than normal cells, allowing PET to visualize areas of abnormal metabolism. The CT component provides precise anatomical localization of these metabolic findings. By integrating metabolic and anatomical information, PET/CT significantly enhances the accuracy of cancer detection, staging, and treatment planning.

In line with the applications of PET/CT in oncology, the true efficacy of PET/CT arises from its ability to combine functional and anatomical information. PET highlights metabolic activity, while CT provides detailed anatomical images. By merging these images, PET/CT offers a comprehensive view with numerous applications in oncology. For example, in tumor detection, increased tracer uptake in PET images typically signals the presence of cancer, helping to differentiate malignant tumors from benign lesions, which usually show lower uptake. PET/CT also plays a critical role in cancer staging, allowing clinicians to determine how far the disease has spread and in prognosis, offering insights into expected patient outcomes. These details are essential for guiding treatment decisions.

Additionally, PET/CT can monitor treatment response by tracking changes in tracer uptake, where reduced uptake after therapy often indicates a positive response. For restaging and recurrence detection, PET/CT enhances the identification of cancer recurrence by highlighting areas of increased metabolic activity, enabling early intervention. In radiation therapy planning, pinpointing metabolically active tumors with PET/CT helps optimize radiation treatment, minimizing damage to surrounding healthy tissues.

#### 3.2.2 Single-Photon Emission Computed Tomography (SPECT)

SPECT is a nuclear imaging technique that creates three-dimensional images of organs and tissues. Unlike conventional X-rays that focus on structural details, SPECT reveals both the anatomy and the functional activity of body parts. This is achieved through radioactive tracing a safe compound injected into the bloodstream that selectively accumulates in specific organs or tissues. As the tracer decays, it emits gamma rays, which are detected by a special camera rotating around the patient. The data from these gamma rays are then reconstructed by a computer to create a 3D model. The brightness of these images depends on how much tracer is absorbed by the tissue, providing insights into its function. SPECT scans are particularly valuable for diagnosing heart diseases, brain disorders like dementia or epilepsy, bone abnormalities, and certain other conditions, as illustrated in Fig. (**9**).

Although PET images offer high sensitivity for cancer detection, SPECT also plays a valuable role in cancer-specific diagnosis. For example, radioiodine assays in SPECT scans are highly effective in imaging thyroid cancer, enabling diagnosis, treatment monitoring, and detection of recurrence. SPECT can also help measure cancerous neuroendocrine tumors of cellular origin of hormonal secretion. Using a specific probe that binds these tumors, SPECT can help diagnose and stabilise them. However, it should be noted that the general application of SPECT in cancer imaging is less extensive compared to PET due to limitations in sensitivity and throughput [52-56].

### 3.3 Non-ionizing Electromagnetic Imaging

Non-ionizing electromagnetic imaging encompasses several techniques that use safe, low-energy electromagnetic waves to produce detailed images of the body’s internal structures. Unlike X-rays and CT scans, which utilize ionizing radiation that can potentially damage cells, these methods do not carry such risks. Consequently, non-ionizing imaging plays a vital role in modern medicine, offering safe and effective diagnostic tools for a wide range of clinical conditions.

#### 3.3.1 Photo and Thermo-acoustic Imaging

Biomedicine has widely adopted lasers because their focused light can produce a range of effects when it interacts with living tissues. These effects include wound healing, blood clotting, vaporization, tissue removal (ablation), and cell death (necrosis). In tissues containing microscopic particles (nanoparticles) that strongly absorb light in the safe range of 650–1350 nm, three specialized light-based therapies, photothermal, photo thermoacoustic, and photoacoustic, can be used. These nanoparticles, which specifically target and respond to light, make them ideal tools for theragnostics, a technique that combines diagnosis and treatment, especially in cancer care. By harnessing Near-Infrared (NIR) light, clinicians can use these nanoparticles to screen, stage, and treat tumors more effectively.

Laser-based photoacoustic imaging and microwave-based thermoacoustic imaging are emerging as powerful tools in biomedicine. These techniques combine the high image contrast of electromagnetic absorption with the excellent resolution of ultrasound imaging. This fusion enables detailed visualization of tissue structure and function, including architecture, physiological and pathological changes, and metabolism. These methods show particular promise for early cancer detection and treatment monitoring. For example, photoacoustic imaging has proven effective in detecting tumor-associated neovascularization in rats [57]. In our research, we have also developed an imaging system to monitor vascular damage during Photodynamic Therapy (PDT), as shown in Fig. (**10**). By providing real-time information on vascular changes, this approach can help guide PDT and other light-based therapies, optimizing treatment by selecting the most effective photosensitizers, light doses, and overall protocols. Looking ahead, the future of photoacoustic and thermoacoustic imaging lies in their potential for functional and molecular imaging. By incorporating targeted contrast agents, these techniques could revolutionize cancer detection and treatment monitoring [58].

When Nanoparticles (NPs) absorb light, they can generate heat in the nearby tissue, a process known as the photothermal effect or hyperthermia. This intense heat can damage proteins, disrupt cell membranes, and kill cancer cells. While very high temperatures (70–80°C) are usually required to directly destroy cancer cells, even lower temperatures can be useful for helping release drugs carried by the NPs. Similarly, photo thermoacoustic and photoacoustic effects involve generating sound waves in the tissue around the NPs [59]. This occurs because the absorbed light energy is converted into heat within the NPs. In photo thermoacoustic therapy, the intense heat (reaching thousands of degrees) causes rapid vaporization of the surrounding liquid, forming tiny gas bubbles that produce sound waves and local fluid movement. In the photoacoustic effect, the heat causes the NP itself to vibrate, creating a sound wave in the surrounding tissue without forming bubbles. This approach is particularly promising for cancer treatment because it minimizes the heating of healthy tissue, while the pressure waves from the hot NPs can still damage cancer cells. Therefore, to develop effective cancer therapies using these techniques, researchers are focused on identifying NPs that work in the photoacoustic regime with Near-Infrared (NIR) light [57-60].

#### 3.3.2 Electrical Impedance Tomography (EIT)

The electrical properties of our bodies vary depending on the type of tissue. This is because tissues consist of cells surrounded by fluids. The cell membrane acts as a barrier that separates the internal fluid from the surrounding fluid. These fluids conduct electricity to different degrees, while the cell membrane behaves like a tiny capacitor and resistor [61].

These electrical properties can be leveraged to create non-invasive images of the body’s internal structures, notably through a technique known as Electrical Impedance Tomography (EIT) (more details are provided in reference [ 35 ]). In EIT, multiple electrodes are placed on the skin’s surface, and small electrical currents are passed through the body. A computer analyzes the resulting voltage measurements to generate two- or three-dimensional images that reflect the body’s internal electrical conductivity.

EIT relies on the principle that different tissues conduct electricity differently—healthy and cancerous tissues, for instance, exhibit distinct electrical properties. Cancerous tissues typically have higher electrical conductivity than healthy ones. This makes EIT particularly promising for early cancer detection, such as in breast cancer screening. The EIT system setup, as shown in Fig. (**11**), enables the detection of these conductivity variations through surface electrodes, offering a non-invasive and radiation-free diagnostic tool [63, 64].

Breast cancer remains a major global health challenge, where early detection is critical for effective treatment. Researchers continue to develop diagnostic tools that are efficient, user-friendly, and safe for patients. This study focuses on evaluating EIT, a promising technique in this field [65, 66]. EIT is a low-cost, non-invasive imaging method that has the potential to complement existing breast cancer detection techniques. The electrical properties of breast tissue have been studied for over 70 years, with encouraging results from laboratory (*in vitro*) measurements. Ongoing research aims to advance both minimally invasive and non-invasive EIT methods for breast cancer screening, offering the promise of affordable, rapid diagnostic options [61-66].

EIT is a non-invasive imaging technique that reconstructs images of the body’s internal conductivity by applying small electrical currents and measuring voltages on the surface of the skin. EIT is gaining recognition for its unique strengths in clinical situations where functional imaging is essential. Unlike imaging methods such as CT, PET, or SPECT, which use ionizing radiation, EIT relies on harmless electrical currents, making it completely safe. This safety advantage is especially important for vulnerable groups, including pregnant women, newborns, and patients requiring long-term monitoring. Additionally, EIT systems are compact and portable, making them well-suited for point-of-care applications in Intensive Care Units (ICUs), outpatient clinics, and low-resource settings [67, 68].

Importantly, EIT provides real-time insights into bodily functions, enabling monitoring of critical conditions like lung function in respiratory patients and heart activity. It can also guide treatments by delivering immediate feedback on their effectiveness. In cancer care, EIT supports early breast cancer detection and helps monitor tumor responses to therapy. Although EIT currently has lower spatial resolution than imaging modalities like CT or MRI, limiting its ability to capture detailed anatomical images, this limitation is less critical in functional and physiological multimodal imaging. Ongoing advances in electrode design, signal processing, and 3D image reconstruction are steadily overcoming these challenges. With its non-invasive nature, real-time imaging capabilities, and strong safety profile, EIT serves as a valuable complementary imaging method, particularly for functional monitoring and rapid clinical assessments.

#### 3.3.3 Near Infrared (NIR) Optical Tomography

As mentioned earlier, cancer is a serious disease characterized by uncontrolled cell growth that can affect any part of the body and spread to other organs. According to the World Health Organization, cancer is the second leading cause of death worldwide, responsible for approximately 10 million deaths annually. By 2040, the number of cancer cases is projected to rise to 30 million, representing nearly a 50% increase [4]. Early detection and effective treatment are essential for managing cancer, particularly in low- and middle-income countries. There is an urgent need for improved methods to identify cancer at an early stage and to accurately assess treatment responses [69].

Near-infrared (NIR) spectroscopy is a technique that detects changes in molecules, such as hemoglobin, water, and fat, within the body. It can also identify specific molecular patterns linked to certain diseases. In cancer diagnosis, NIR spectroscopy differentiates healthy tissue from cancerous tissue by analyzing how these molecules absorb near-infrared light [59]. Although NIR does not provide detailed images of deep tissues, it offers a fast and affordable way to assess blood flow and other characteristics at the level of the smallest blood vessels. By examining these factors, NIR helps distinguish tumors from healthy tissue [69]. Early cancer detection is vital for successful treatment, as illustrated in Fig. (**12**). Currently, various imaging techniques like X-rays and PET scans are used for cancer diagnosis. While advanced methods, such as CT scans and MRIs, exist, they may not always be accessible or suitable for every patient [23-30].

Near-infrared spectroscopy is a powerful technique used to analyze a sample’s composition by directing near-infrared light onto it. When near-infrared light interacts with molecules, different molecular groups vibrate at specific frequencies, much like springs oscillating at their natural rhythm. These vibration frequencies depend on the types of atoms, bond strengths, and the surrounding molecular environment. Functional groups such as carbonyls, hydroxyls, and amines have distinct structural characteristics, causing them to vibrate at unique, identifiable frequencies. By measuring these vibrations, NIR spectroscopy offers valuable insights into the molecular composition of a sample [ 68 ].

Infrared (IR) spectroscopy, a related technique, uses infrared light to excite molecular vibrations as well. Molecules absorb IR light at frequencies that correspond to specific atomic movements. These vibrations represent subtle internal motions within the molecule. However, not all molecular vibrations are detectable with IR spectroscopy, as only those that produce a change in dipole moment are “IR-active”—a limitation governed by selection rules in molecular spectroscopy [ 70 ].

Near-infrared light has higher energy than regular infrared light, enabling NIR spectroscopy to detect additional molecular vibrations that are not visible with conventional IR. These vibrations are combinations of simpler ones, similar to how a musical chord is made up of several notes. By analyzing these combined vibrations, NIR can accurately identify and quantify different chemical groups within a sample. NIR spectroscopy is valuable because it is fast, painless (non-invasive), and applicable to various sample types, including liquids, solids, and gases. It is also non-destructive, meaning it does not damage the sample. However, interpreting NIR data requires complex calculations to relate the absorbed light to the sample’s properties [71]. Infrared imaging, on the other hand, is a non-invasive technique that captures thermal radiation emitted by objects, including the human body, to generate temperature-based images. While useful for detecting surface temperature variations, infrared imaging differs significantly from advanced techniques like EIT, PET, CT, and MRI in terms of principles, capabilities, and clinical applications.

The underlying principles vary significantly among these imaging modalities. Infrared imaging works by measuring emitted infrared radiation, which corresponds to surface temperature, limiting its ability to penetrate deeper tissues. In contrast, Electrical Impedance Tomography (EIT) measures electrical conductivity to reconstruct images of internal tissues. Positron Emission Tomography (PET) detects gamma rays emitted by radiotracers to visualize metabolic activity, while Magnetic Resonance Imaging (MRI) uses magnetic fields to generate detailed anatomical and functional images. A key difference lies in imaging depth. Infrared imaging is restricted to surface-level information, limiting its use for examining deeper structures or functions. EIT can penetrate tissues to detect changes in conductivity, providing functional images of internal organs. PET and MRI offer high-resolution imaging for both superficial and deep tissues. Regarding the type of information provided, infrared imaging mainly detects surface temperature changes, useful for fever screening and infection detection. EIT captures physiological processes, such as lung ventilation. PET delivers metabolic data critical for cancer detection, and MRI provides exceptional anatomical detail alongside functional insights, which is particularly valuable for brain research.

### 3.4 Emerging Techniques in Cancer Imaging

Doctors are gaining new tools in the fight against cancer, with innovative imaging techniques being developed to enhance diagnosis and treatment. One promising method uses a special type of light emitted by radioactive tracers, offering a potentially faster and more affordable alternative to traditional PET scans. Advances in MRI technology also allow doctors to visualize blood flow and activity within tumors in greater detail, aiding both diagnosis and treatment monitoring. These innovations represent just a few examples of how emerging technologies are transforming cancer imaging, paving the way for earlier detection, improved therapies, and, ultimately, better outcomes for patients [70].

Among these advances, Cerenkov imaging stands out as a novel technique that captures the faint glow produced by radioactive tracers similar to those used in PET scans to create images of tumors. This approach could provide a quicker, less expensive option for cancer screening, particularly in resource-limited settings or for patients who require frequent monitoring during treatment [71]. However, Cerenkov imaging is still in its early stages. Researchers are working to develop more sensitive cameras capable of capturing this weak light and producing images with clarity comparable to PET scans. Despite these challenges, Cerenkov imaging holds great promise for making cancer diagnostics more accessible and improving patient care in the future [72].

Cancer imaging is on the brink of a major breakthrough thanks to exciting new technologies. Artificial Intelligence plays a central role, with computers learning from vast collections of scans. This enables AI to automatically detect cancer, analyzing images much faster than humans and identifying tiny details that might otherwise be missed. This could lead to earlier and more accurate cancer detection, even at the earliest stages. However, AI systems require continuous training to maintain accuracy, and the large amounts of data involved raise important patient privacy concerns.

Despite these challenges, the future of cancer imaging looks very promising. The combination of AI’s analytical power with emerging techniques like Cerenkov imaging has the potential to revolutionize cancer diagnosis and treatment, offering hope for a much brighter future for patients [73].

## 4 CLINICAL APPLICATIONS OF CANCER IMAGING

Imaging plays a vital role throughout every stage of cancer care, from the first symptoms to ongoing management. During initial diagnosis, techniques such as X-rays, CT scans, and MRIs provide valuable information by detecting suspicious lumps or masses, pinpointing tumor location, and estimating size and spread. This information is crucial for deciding whether a biopsy is necessary and helps confirm the cancer diagnosis. Once cancer is diagnosed, staging and determining the extent of spread becomes essential for treatment planning. Imaging methods like PET scans, which highlight areas of increased metabolic activity, assist in identifying whether cancer has spread to lymph nodes or other organs [74]. These detailed images empower doctors to choose the most effective treatment options, including surgery, radiation, or chemotherapy, as depicted in Fig. (**13**). Finally, multimodal imaging remains indispensable during treatment and follow-up care. Regular scans are used to monitor how well the treatment is working, assess tumor shrinkage, and detect any potential recurrence. This continuous monitoring allows doctors to adjust treatment plans as needed, ensuring the best possible outcomes for patients [75].

### 4.1 Early Detection and Cancer Screening

Early detection is a cornerstone in the fight against cancer. Cancer screening uses various imaging and testing methods to identify cancer before symptoms appear, often making it more treatable. Screenings such as mammograms for breast cancer or colonoscopies for colorectal cancer can detect precancerous cells or tumors at an early stage. Early detection offers many benefits. Treatments tend to be less invasive and more successful at this point, leading to better patient outcomes and even potential cures. However, it is important to remember that not all cancers currently have effective screening methods. Additionally, some screening tests carry risks and limitations, so discussing the pros and cons with your doctor is essential, as illustrated in Fig. (**14**) [76].

### 4.2 Tumor Characterization and Analysis

Tumor characterization and analysis are closely linked steps in the fight against cancer, as illustrated in Fig. (**15**). While diagnosis confirms the presence of cancer, characterization provides a detailed understanding of the specific cancer type and its aggressiveness. This precise information is essential for designing an effective treatment plan [3, 4]. Diagnosis often begins with imaging techniques, such as X-rays, CT scans, and MRIs, which help determine the tumor’s location, size, and sometimes its function. However, these methods alone may not offer enough detail to identify the exact type of cancer. That is why when biopsies come into play—tissue samples taken from the tumor are examined under a microscope to determine the exact cell type and assess how aggressive the cancer is [23-35].

Beyond traditional evaluation, advancements in molecular profiling are playing an increasingly important role. These techniques analyze the tumor’s genetic makeup, identifying mutations or specific markers that offer vital insights into the cancer’s behavior and its likely response to treatment. This information enables doctors to move beyond a one-size-fits-all approach and customize therapies to target the unique weaknesses of the cancer cells [8]. In essence, tumor characterization and diagnosis work hand-in-hand to create a complete picture of the cancer, empowering doctors to develop the most precise and effective treatment plans possible.

### 4.3 Cancer Staging and Treatment Plans

The coordinated process of cancer staging and treatment planning determines the best course of action for each patient. Staging involves classifying the severity of cancer based on its extent. This typically includes a combination of imaging techniques, such as CT scans, PET scans, and sometimes biopsies, to assess the tumor’s size, spread to lymph nodes, and the presence of distant metastases [35]. Understanding the stage is critical for developing an effective treatment plan. Early-stage cancers, confined to one area, can often be successfully treated with surgery alone. More advanced stages, where cancer has spread, usually require a multi-faceted approach. This may involve surgery to remove the primary tumor, followed by radiation therapy to eliminate any remaining microscopic cancer cells in the area. Chemotherapy, a systemic treatment delivered through medication, is often used to target cancer cells that have spread beyond the original site [76]. The specific treatment plan is carefully tailored based on the cancer type, stage, and the patient’s overall health. Factors like age, underlying medical conditions, and personal preferences also play important roles in decision-making. This collaborative, individualized approach guided by staging insights ensures that each patient receives the most effective and personalized treatment possible.

### 4.4 Treatment Monitoring and Response Assessment

Monitoring and response evaluation are essential throughout cancer treatment to assess its effectiveness and make timely adjustments if needed. Imaging methods, such as CT scans, MRIs, and PET scans, play a vital role in this process. By comparing scans taken before, during, and after treatment, doctors can track changes in tumor size and activity. Tumor shrinkage or decreased metabolic activity usually indicates a positive response, while stable or growing tumors may require a change in therapy [77]. In addition to traditional imaging, minimally invasive methods like liquid biopsies are becoming increasingly important. These tests analyze circulating tumor DNA released into the bloodstream by cancer cells. Tracking changes in this DNA over time enables doctors to monitor treatment response and detect potential recurrences earlier [78]. The information gathered from monitoring and response assessment is critical for making informed decisions. If a treatment is ineffective, it can be discontinued or replaced with a more appropriate option. This adaptive approach ensures that patients receive the best possible care throughout their cancer journey [78, 79].

### 4.5 Experimental Work: Multimodal Therapy

Multimodal therapy is a cancer treatment strategy that combines multiple therapeutic methods to target cancer from different angles. This approach typically involves a combination of surgery, chemotherapy, and radiation therapy. Multimodal therapy is applied to various cancer types and often proves more effective than any single treatment alone, as illustrated in Fig. (**16**). Beyond advances in imaging, ongoing cancer research continues to explore innovative treatments and preventive measures through experimental studies [79–84].

Researchers are harnessing the body’s natural immune system to combat various types of cancer. Techniques, such as CAR T-cell therapy modify a patient’s immune cells to better recognize and attack cancer cells. Immune checkpoint inhibitors work by releasing the brakes on the immune system, enabling a stronger and more sustained immune response against cancer. These immunotherapies hold great promise for achieving long-term remission and, in some cases, potential cures.

Below are some of the most promising strategies under investigation:

#### 4.5.1 Targeted Therapies

Cancer cells often carry unique genetic mutations that drive their growth. Targeted therapies are drugs designed to exploit these specific mutations, launching a customized attack on the cancer’s weak points. This approach aims to minimize side effects by precisely focusing on the vulnerabilities of cancer cells.

#### 4.5.2 Nanotechnology Applications

The emerging field of nanotechnology offers promising new avenues for cancer treatment. Nanoparticles can be engineered to deliver drugs directly to tumors, reducing damage to healthy tissues. Moreover, nanoparticles can assist in imaging, helping to visualize tumors and monitor treatment responses more effectively.

#### 4.5.3 Gene Editing Techniques

CRISPR, a powerful gene-editing tool, is being investigated for its ability to precisely target the genetic mutations that drive cancer development. Although still in the early stages, CRISPR holds promise as a potential one-time treatment for certain types of cancer.

#### 4.5.4 Oncolytic Viruses

These are specially engineered viruses designed to infect and destroy cancer cells. Acting like Trojan horses, they infiltrate tumors, replicate within them, and ultimately cause tumor destruction. Oncolytic viruses offer a targeted and potentially adaptable approach to cancer therapy.

It is important to note that these innovative methods are still under research, with many remaining in preclinical phases. Nevertheless, their potential is significant, providing hope for a future where cancer is not just managed but prevented, effectively treated, or even cured [78-84].

### 4.6 Results & Analysis

To date, cancer remains a major global health challenge with alarming statistics. The World Health Organization (WHO) reports approximately 5.7 million cancer deaths among men and 4.5 million among women annually [4]. According to the World Cancer Research Center, global cancer incidence has surged dramatically, rising from 12.7 million cases in 2010 to 20.5 million in 2023. The impact varies by region. In the United States, lung, breast, prostate, and colon cancers are the most prevalent, as reported by the CDC. China records the highest number of new cancer cases each year, particularly liver, stomach, and esophageal cancers, according to the National Cancer Institute of China. In India, breast, ovarian, and oral cancers are increasingly common, highlighted by the Indian Council of Medical Research. Europe continues to experience high rates of lung, breast, and colorectal cancers, largely driven by smoking and alcohol consumption, as noted by the European Cancer Reporting System.

Major cancer risk factors differ across regions: in the US, tobacco use, obesity, diet, and physical inactivity are predominant; in China, tobacco, chronic infections (such as HBV and HCV), diet, and environmental pollution play key roles; in India, tobacco use (both smoking and chewing), infections (especially HPV related to cervical cancer), dietary habits, and alcohol consumption are significant; and in Europe, tobacco, alcohol, diet, obesity, and occupational exposures are the primary contributors, as summarized in Table **2**.

A survey of multimodality imaging techniques from 2014 to 2023 reveals significant advances and shifts in clinical practice. In 2014, CT scans dominated imaging services, accounting for 40%, followed by MRI at 30% and PET scans at 15%. By 2023, MRI became the most commonly used modality, representing 48% of cases, surpassing CT at 31% and PET at 30%. This shift reflects the growing preference for MRI, driven by its higher resolution and enhanced imaging capabilities. The availability of PET technology also increased steadily, rising from 55% of facilities in 2014 to 77% by 2023. Notable technological advances include the introduction of advanced PET scanners in 2014, improved MRI resolution in 2015, and hybrid scanners, such as PET/MRI systems emerging in 2017. More recently, AI-enhanced imaging analysis and portable imaging devices have been introduced, with significant progress in AI and machine learning integration occurring in 2023. These developments highlight a trend toward more accurate, accessible, and high-quality imaging technologies, as summarized in Table **3** .

In a comparative study of multimodality imaging for cancer detection and management, each technique offers distinct advantages and limitations. X-ray radiography is inexpensive and widely accessible, making it effective for detecting fractures and calcifications; however, it provides limited soft tissue contrast and involves a low dose of radiation. CT scans deliver high-resolution images of bones, soft tissues, and blood vessels, which are critical for accurate cancer staging and treatment planning. Despite these benefits, CT scans are costly and expose patients to relatively high radiation doses, making them unsuitable for pregnant women and less ideal for frequent monitoring.

MRI employs strong magnetic fields and radio waves to produce excellent soft tissue contrast without ionizing radiation, making it ideal for detailed tumor imaging. Nevertheless, MRI is expensive, time-intensive, and may be contraindicated for patients with certain implants. Ultrasound offers real-time imaging, is safe for pregnant women, and is often used for repeated monitoring due to its lack of ionizing radiation. Its limitations include restricted tissue penetration and operator-dependent image quality. PET scans detect tumors based on their metabolic activity and are valuable for cancer staging. However, they are expensive and require a cyclotron to produce radioactive tracers. Similarly, SPECT scans generate 3D images to assess specific functions such as blood flow and bone health but are generally less comprehensive and accessible than PET scans and involve a small radiation dose. Each imaging modality plays a crucial role in cancer diagnosis, with the choice depending on the clinical context and patient-specific considerations, as summarized in Table **4**.

Cancer care research explores patient demographics, cancer types, treatments, outcomes, and resource utilization. The average age of cancer patients is around 55 years, ranging from 30 to 85 years, with women comprising about 60% of cases and men 40%. Socioeconomic factors also significantly impact care, as nearly half of patients live below the poverty line, limiting their access to treatment. The most common cancers include breast, lung, and prostate cancer, with survival rates and outcomes varying widely depending on the cancer type and stage at diagnosis—early detection generally leads to a better prognosis.

Treatment approaches vary among patients: on average, patients undergo six chemotherapy cycles; targeted therapies are administered to about 30% of patients, primarily those with specific genetic mutations; and immunotherapy, though still emerging, shows a response rate in approximately 25% of patients, with 40% of responders experiencing favorable outcomes. For example, one-year survival rates can reach up to 80%, while five-year survival rates may be as high as 50%. Quality of life is reported positively by 70% of patients, although it can be impacted by treatment side effects and disease progression. A recurrence rate of 20% underscores the critical importance of early diagnosis. Healthcare utilization data indicate an average of 500 hospital admissions, with a typical stay lasting four days. Treatment costs average $50,000 per patient but vary widely depending on treatment type and insurance coverage, highlighting the significant financial burden faced by patients, as detailed in Table **5** .

Global cancer statistics from 2014 to 2023 reveal a concerning rise in both new cases and deaths. Annual cancer deaths increased from 8.2 million in 2014 to 10.7 million by 2023. The most common cancers worldwide are lung, breast, and colorectal cancers. Meanwhile, the availability of radiotherapy services in hospitals steadily improved, rising from 60% in 2014 to 82% by 2023. This growth reflects ongoing efforts to enhance access to cancer care; however, the rising incidence and mortality rates highlight the urgent need for continued global health initiatives. The COVID-19 pandemic in 2020 significantly disrupted cancer care, contributing to a sharp increase in deaths and new cases that year, as detailed in Table **6** .

The Cancer Services Analysis Table for mortality statistics by sex from 2014 to 2023 reveals concerning trends in cancer mortality. Since 2014, cancer has claimed the lives of 4.3 million men and 3.3 million women, totaling 7.6 million deaths. Mortality rates for both men and women have steadily increased each year. By 2023, male deaths are projected to reach 5.7 million and female deaths 4.5 million, bringing the total to 10.2 million. This persistent rise highlights a consistent gender disparity, with males experiencing higher mortality throughout the period. The impact of the COVID-19 pandemic is clearly reflected in 2020, as shown in Fig. (**17**), which documents a sharp increase in mortality alongside a decline in cancer care and diagnosis. Mortality rates continued to climb after the pandemic, culminating in the recorded deaths of 2023. These data underscore the urgent need for enhanced cancer prevention, early detection, and improved therapeutic strategies to better manage the increasing mortality rate, as summarized in Table **7** .

The Cancer Services Analysis table tracking patient populations by gender from 2014 to 2023 reveals a significant and steady rise in cancer diagnoses. In 2014, there were 6.5 million male patients and 5 million female patients, totalling 11.5 million cases. This number has increased annually, reflecting the growing burden of cancer. By 2019, the total reached 14 million patients, including 8 million men and 6 million women. The COVID-19 pandemic further accelerated this trend in 2020, with diagnoses rising to 14.8 million patients, as illustrated in Fig. (**18**). This upward trajectory continued in the following years, reaching 16.5 million cancer patients by 2023, including 9.5 million men and 7 million women, as detailed in Table **8**. These figures underscore a consistent and substantial rise in cancer incidence, emphasizing the urgent need for enhanced prevention, early detection, and treatment strategies to manage the increasing patient load effectively.

The Cancer Services Analysis, as shown in Table **9** , detailing the main causes by region from 2014 to 2023, highlights persistent regional risk factors contributing to cancer incidence and mortality. In the United States, lifestyle-related factors, such as tobacco use, obesity, poor diet, and physical inactivity, remain major contributors to cancer, despite ongoing public health efforts. This underscores the continued need for robust prevention initiatives. In China, high cancer rates are primarily linked to tobacco use, chronic infections such as hepatitis B and C (HBV and HCV), poor nutrition, and environmental pollution, with liver and stomach cancers being especially prevalent. Strengthening infection control measures and improving environmental health are essential to addressing these challenges. In India, increasing cancer incidence is associated with tobacco use (both smoking and chewing), infections (notably HPV), and dietary habits, raising particular concern for cervical and oral cancers. Expanding HPV vaccination programs and public awareness campaigns are critical for prevention. Europe consistently reports high rates of lung, colorectal, and other cancers, largely driven by tobacco and alcohol use, poor diet, obesity, and occupational exposures. While interventions are in place, the data emphasize the need for intensified efforts focused on lifestyle modification and risk reduction strategies, as summarized in Table **9** .

Cancer continues to pose a significant global health challenge, with India experiencing a particularly sharp rise in new cases. In 2022 alone, India reported over 1.4 million new cancer cases, highlighting the growing burden of the disease. Among women, breast cancer remains the most common type, while lung and oral cancers are predominant in men, as illustrated in Fig. (**19**). These patterns align with global trends, where cancer rates continue to increase. According to the World Health Organization, millions of new cancer cases are expected worldwide each year, as shown in Fig. (**20**), underscoring the urgent need to strengthen prevention, early detection, and treatment efforts to combat this devastating illness.

The effects of radiation exposure depend on whether it is acute (short-term) or chronic (long-term), with acute exposure typically causing immediate symptoms and chronic exposure posing cumulative risks. Prolonged exposure to low doses exceeding 100 mSv over a lifetime can increase the likelihood of cancer. For moderate radiation doses, medical interventions, such as supportive care, antibiotics, and bone marrow transplants, can mitigate the effects and improve recovery. As shown in Table **10**, the effects of radiation on the human body are organised by the radiation dose range (measured in millisieverts, mSv) and associated disorders.

Medical multimodality imaging techniques differ significantly in radiation exposure and associated safety considerations. Non-ionizing methods, such as ultrasound and MRI, are safe for routine use since they do not involve ionizing radiation. Low-dose techniques, including X-rays and mammography, use minimal radiation and are generally considered safe for periodic screening. In contrast, high-dose modalities like CT scans, PET scans, and fluoroscopy expose patients to higher levels of radiation and are therefore used only when medically necessary. Repeated imaging with CT and PET can lead to cumulative radiation doses, slightly increasing the long-term risk of cancer. For pregnant patients, non-ionizing imaging methods are preferred to avoid potential risks to the fetus from ionizing radiation. Table **11** summarizes typical radiation doses for various imaging modalities and their potential effects on the human body.

## 5 DISCUSSION

Multimodal imaging is revolutionizing cancer care by integrating data from X-ray, CT, MRI, PET, and ultrasound scans to create a more comprehensive picture of the disease. This approach enables more accurate diagnosis, precise staging, and effective monitoring of treatment response. The benefits of multimodality imaging include improved accuracy, personalized treatment plans, and potentially less invasive procedures. However, challenges such as cost, radiation exposure, and complex data interpretation remain. Advances in AI, novel biomarkers, and theranostics offer promising improvements, paving the way for a brighter future in cancer imaging.

The fight against cancer extends beyond advanced imaging techniques. Experimental research is exploring innovative methods for prevention, diagnosis, and treatment. Immunotherapy, including CAR T-cell therapy and immune checkpoint inhibitors, leverages the body’s immune system to target cancer with remarkable precision. Targeted therapies, such as those used for HER2-positive breast cancer, act like personalized weapons aimed at specific genetic mutations, reducing side effects. Nanotechnology presents exciting opportunities by using nanoparticles to deliver drugs directly to tumors or to enhance imaging and treatment monitoring. Gene-editing technologies like CRISPR offer the potential for one-time cures by correcting genetic mutations that drive cancer growth. Additionally, oncolytic viruses engineered to selectively infect and destroy cancer cells provide a promising path toward precise and adaptable treatments. Though still in early development, these experimental approaches hold enormous potential to transform cancer from a formidable adversary into a disease that can be prevented, effectively treated, or even cured.

Consider a 55-year-old woman experiencing a persistent cough and unintended weight loss. A chest X-ray reveals a suspicious lung nodule, prompting a follow-up CT scan that provides a more detailed view. However, a CT scan alone cannot definitively distinguish between cancerous and benign lesions. To assess metabolic activity and potential spread, a PET scan is performed. Increased uptake in the nodule on the PET scan raises the suspicion of cancer. The next critical step is a biopsy to obtain tissue samples for microscopic examination, providing a definitive diagnosis. This case illustrates how multimodality imaging, combining X-ray, CT, and PET scans, works synergistically to offer a comprehensive view, enabling accurate diagnosis and guiding personalized treatment planning.

In parallel, deep learning has emerged as a transformative tool in colorectal cancer (CRC) diagnosis by significantly improving the accuracy and efficiency of histopathology image analysis. Recent studies demonstrate that deep learning algorithms can classify and diagnose CRC with remarkable precision, often outperforming traditional methods. These advances enable the automated detection of subtle pathological features, reducing diagnostic variability and boosting early detection rates. Integrating deep learning into clinical workflows holds promise to support pathologists in decision-making, streamline processes, and ultimately enhance patient outcomes. However, further validation across diverse datasets and careful clinical implementation remain essential for its widespread adoption.

## 6 CHALLENGES AND FUTURE DIRECTIONS

**A. Challenges:**

**Accessibility and Cost:** Advanced imaging techniques like PET scans and MRIs can be expensive and require specialized equipment, limiting access in resource-restricted settings [79].

**I) Radiation Exposure:** Techniques like CT scans and X-rays involve ionizing radiation, elevating worries about the capability of long-term results, especially for frequent tracking.

**II) Specificity and Accuracy:** While imaging plays a crucial role, some techniques may struggle to differentiate between cancerous and benign lesions, necessitating further tests for definitive confirmation.

**III) Data Management and Interpretation:** The sizable quantity of statistics generated by using complicated imaging techniques may be challenging to control and interpret effectively.

**B. Limitations and Future Directions:**

This section offers a thorough analysis of the challenges and opportunities related to multimodality imaging in cancer treatment. A major limitation is the variability in imaging protocols across different institutions, which results in inconsistent data quality and makes comparing outcomes difficult. Additionally, many studies reviewed are retrospective, raising concerns about potential biases and limiting the ability to draw definitive causal conclusions. The section also discusses publication bias, where positive findings are more likely to be published, potentially skewing the overall perception of imaging effectiveness.

To overcome these challenges, future research should prioritize prospective and multimodality studies to validate findings and ensure broader applicability, as outlined in the following points. Additionally, integrating AI and machine learning for real-time data analysis holds immense potential to improve diagnostic accuracy, streamline workflows, and support personalized treatment strategies. By addressing these limitations and pursuing these future directions, the field of multimodal imaging can achieve more reliable, efficient, and impactful advances in cancer treatment.

**C. Current Imaging Strategies and Associated Risks**

I) **Radiation Dose:** Imaging techniques such as CT scans and X-rays use ionizing radiation to produce detailed images. Although the radiation dose from a single scan is generally low, cumulative exposure, especially in patients undergoing frequent monitoring or in younger individuals, raises concerns about long-term risks. Therefore, a careful risk-benefit assessment is essential when selecting these imaging methods [79-83].

II) **Cost:** Advanced imaging modalities like PET scans and MRIs are often expensive due to the high costs of equipment, tracer production (in PET scans), and the need for specialized personnel. These financial barriers can restrict access in resource-limited settings, contributing to disparities in healthcare availability.

**III) Specificity and Accuracy:** While imaging is vital for cancer detection, some methods may not always clearly distinguish between malignant and benign lesions. This limitation can lead to false positives, causing unnecessary anxiety and additional invasive tests, such as biopsies. Conversely, false negatives may delay diagnosis and treatment, impacting patient outcomes.

**IV) Data Overload:** Complex imaging technologies generate vast amounts of data, posing challenges in data management, storage, and interpretation. Effective handling of this information requires advanced data management systems and trained professionals to analyze and convert it into actionable clinical insights.

**V) Limited Tissue Characterization:** Some imaging techniques, such as X-rays and ultrasounds, primarily provide anatomical information. While valuable for initial detection and staging, they often lack the ability to reveal detailed information about the specific cancer type or its aggressiveness. This limitation usually necessitates additional procedures like biopsies for precise characterization.

Nonetheless, the future of cancer imaging is bright with innovation, focused on overcoming current limitations and personalizing patient care. Some exciting directions shaping this future are outlined below:

- **AI-driven Imaging Approaches:** The era of one-size-fits-all imaging protocols may soon be behind us. Advances in artificial intelligence are enabling personalized imaging strategies. Machine learning algorithms trained on extensive patient data and clinical images can predict how an individual’s tumor will respond to different imaging methods. This innovation allows for tailored scans that focus on acquiring the most relevant information for each patient’s unique condition—possibly opting for less expensive or radiation-free techniques that still provide sufficient diagnostic detail. Moreover, Artificial intelligence algorithms are being continuously refined to enhance accuracy, automate evaluation, and potentially perceive diffused patterns invisible to the human eye, leading to earlier diagnoses and personalized treatment plans [79–83].
- **Theranostics:** Theranostics is an emerging concept that integrates diagnostic and therapeutic functions into a single agent. These agents, often nanoparticles or molecules, target specific biomarkers in cancer cells. They not only illuminate tumors during imaging (diagnosis) but also deliver therapeutic payloads directly to cancer cells. This targeted approach holds significant promise for minimizing side effects on healthy tissues while simultaneously enabling real-time monitoring of treatment response through imaging.
- **Advanced Contrast Agents:** Researchers are continually developing new contrast agents with enhanced capabilities. The development of new contrast agents with improved sensitivity and specificity is an ongoing area of research. These agents would not only clearly highlight tumors but also provide detailed information about the cancer type or its aggressiveness. These next-generation agents could streamline the diagnostic process and help guide treatment decisions tailored to the specific cancer profile. Further, they aim to provide clearer differentiation between cancerous and healthy tissues.
- **Radiomics and Machine Learning:** Radiomics involves extracting quantitative features from medical images. By inputting this data into machine learning algorithms, researchers are unlocking new layers of information from existing scans. This enhanced data can improve cancer prognosis, predict treatment responses, and identify patients at higher risk of recurrence. Hence, extracting quantitative data from medical images and applying machine learning algorithms to analyze this information is a rapidly growing field with the potential to enhance diagnosis, treatment planning, and response prediction.
- **Multimodality Imaging:** Combining exceptional imaging strategies, like PET/CT scans, is becoming increasingly more commonplace, supplying a greater comprehensive picture of cancer for higher analysis, staging, and remedy-making plans.
- **Emerging Techniques:** Innovative imaging methods like Cerenkov imaging and photoacoustic imaging are being explored for their potential to provide faster, more affordable, or more portable alternatives to current technologies. Cerenkov imaging utilizes faint light emitted by radioactive tracers, offering a potentially quicker and less expensive option compared to PET scans. Photoacoustic imaging uses light pulses to generate sound waves in tissues, enabling high-resolution imaging of cancers near the surface of the skin.

These are just glimpses into the exciting future of cancer imaging. By leveraging the power of AI, theranostics, nanotechnology [84], and advanced digital tools [85, 86], researchers in blood-based medicine and imaging are poised to revolutionize cancer diagnosis and treatment, ultimately leading to more personalized and effective care for patients [87, 88].

## CONCLUSION

This review explores the transformative impact of multimodality imaging in cancer care, providing a comprehensive framework for diagnosis, treatment planning, and monitoring. Key highlights include the integration of advanced technologies, such as PET/MRI fusion, radiomics, and hybrid imaging, which collectively improve diagnostic accuracy, tumor characterization, and personalized treatment approaches.

Moreover, recent advances in cancer diagnosis and treatment offer renewed hope. Cutting-edge imaging techniques, including AI-driven methods, provide a more comprehensive view of tumors, enabling more accurate diagnoses and personalized treatment plans. Breakthroughs in immunotherapy and targeted therapies are expanding options for patients who previously had limited choices.

These innovations have led to better patient outcomes by enabling earlier detection, precise staging, and dynamic monitoring of therapy response. Looking forward, future research should prioritize the development of AI-driven predictive analytics, the expansion of theranostic applications combining diagnosis and therapy, and the optimization of hybrid imaging systems for wider clinical use. In the coming decade, multimodality imaging is set to revolutionize clinical oncology by bridging the gap between research and practice, ultimately delivering more effective, personalized, and accessible cancer care.

Furthermore, the future holds great promise; artificial intelligence is set to transform the analysis of complex imaging data, while gene editing technologies like CRISPR offer the potential for lasting cures. The ongoing quest for innovation in cancer research points toward a future where cancer can not only be effectively managed but potentially prevented or eradicated altogether.

## Figures and Tables

**Fig. (4):**
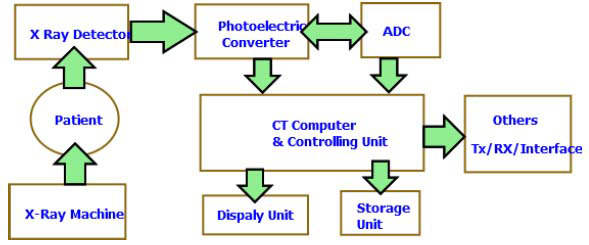
Methodology or working layout of the X-Ray imaging techniques.

**Fig. (7):**
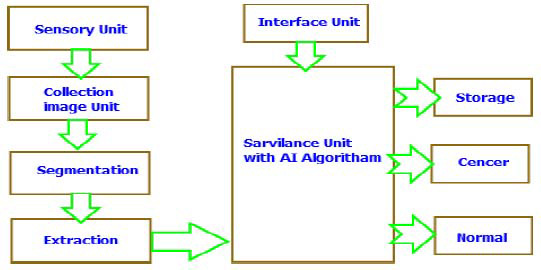
Block diagram of mri imaging techniques (It uses strong magnetic fields and radio waves to generate detailed images of the object's internal structures).

**Fig. (8):**
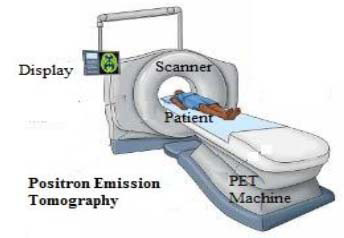
PET imaging techniques broken down into key components and steps.

**Fig. (9):**
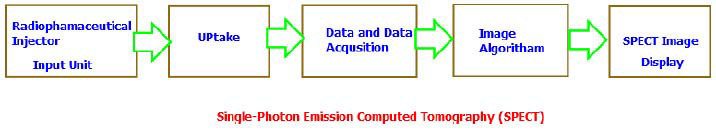
The functional processes of the SPECT imaging techniques.

**Fig. (10):**
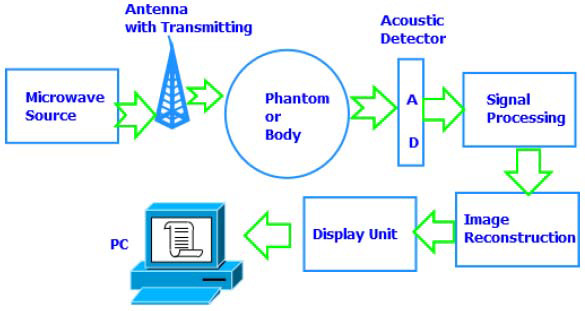
Block diagram of photo and thermo-acoustic imaging techniques.

**Fig. (11):**
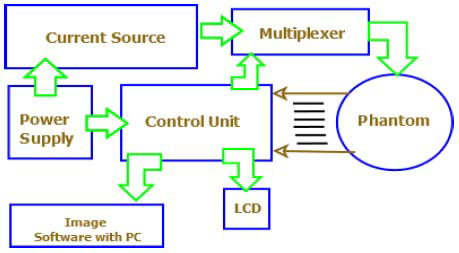
Block diagram of EIT imaging techniques outlining the key components and process.

**Fig. (12):**
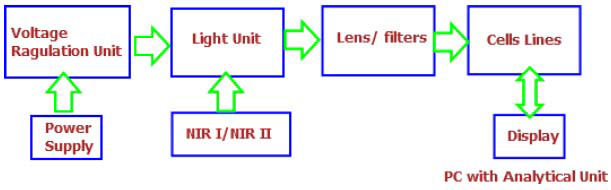
Near-infrared optical tomography imaging techniques involve a process divided into key components and steps.

**Fig. (13):**
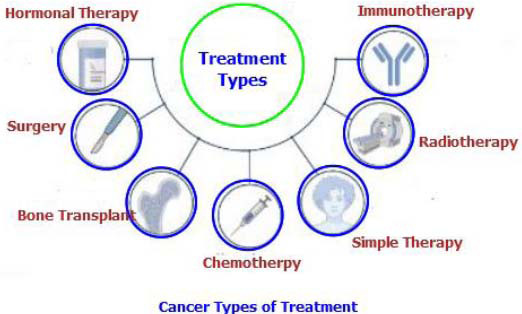
Innovative cancer therapies: Types and transformations.

**Fig. (14):**
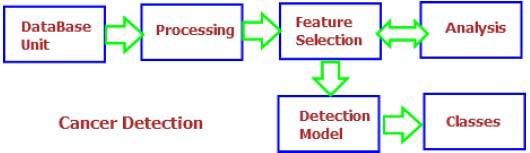
Identifying cancer: Advances in detection technologies.

**Fig. (15):**
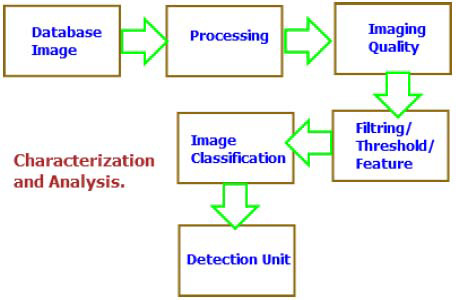
Characterization and analysis of multimodal cancer treatments.

**Fig. (16):**
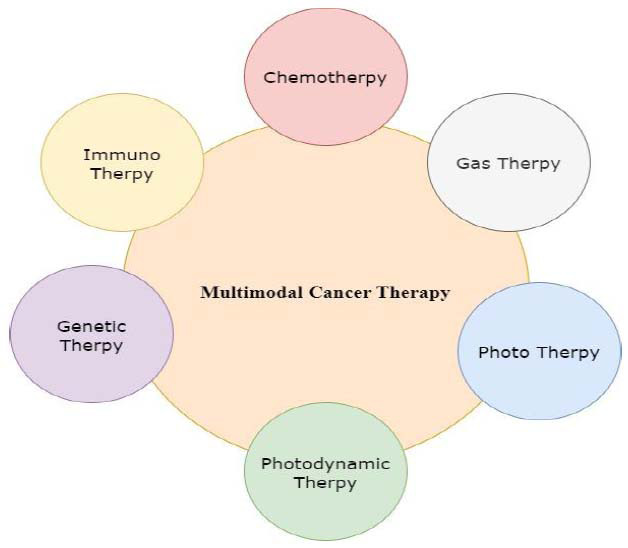
Multimodal therapy redefines cancer treatment.

**Fig. (17):**
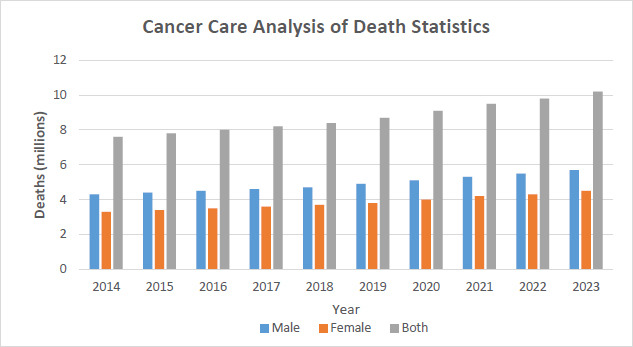
Cancer care analysis of death statistics.

**Fig. (18):**
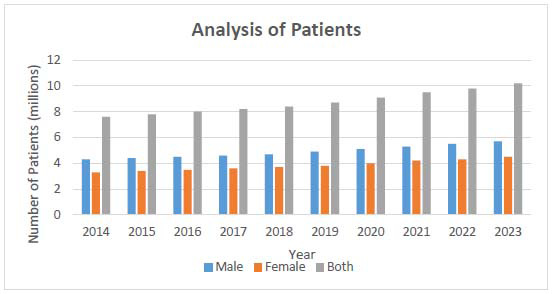
Cancer care analysis of number of patients.

**Fig. (19):**
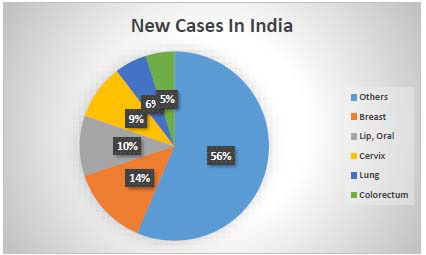
New case in India in both sexes.

**Fig. (20):**
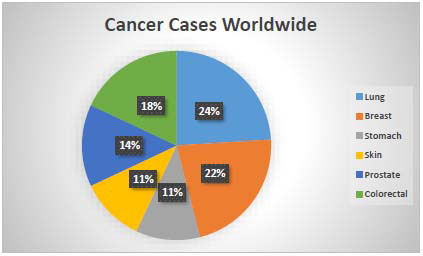
New cases worldwide in both sexes.

**Table 1:** A Literature review according to multimodality imaging in cancer care.

**S.No.**	**Author and Year\Refs.**	**Applications**	**Cancer Care Types**	**Related Description**
1	He *et al.* 2020 [13]	Lung Cancer Screening	Early Detection	Increased sensitivity and identifying potentially malignant lesions at earlier stages.
2	K. Pinker *et al.* 2022 [14]	Breast Cancer	Early Detection	This study is based on sensitivity and specificity for identifying potentially malignant lesions at earlier stages.
3	Prasad *et al.* 2024 [15]	Breast Cancer Imaging	Diagnosis and Tumor Characterization	Provided more accurate differentiation between benign and malignant tumors and also provided insights into tumor characteristics.
4	Pujana-Vaquerizo, *et al.* 2017 [16]	Prostate Cancer	Diagnosis and Tumor Characterization	Evaluated tumor aggressiveness, size, and other characteristics, aiding diagnosis and treatment planning.
5	Bertolini *et al.* 2023 [6]	Optical Imaging for Tumor Therapy	Treatment Planning	Enhanced visualization of tumor extent, surrounding anatomy, and potential target interventions.
6	Jennifer *et al.* 2022 [17]	Head and Neck Cancer	Treatment Planning	The potential targets for surgery, radiation therapy, or other interventions with precise treatment planning
7	IH Kunkler *et al*. 2023 [9]	Breast Cancer Imaging	Treatment Monitoring	Assessed treatment response by monitoring changes in tumor size, metabolism, and other parameters.
8	van der *et al.* 2021 [18]	PET Imaging for Treatment Monitoring	Treatment Monitoring	Multimodality imaging can help identify early signs of treatment resistance, allowing for adjustments to the treatment plan.
9	Ghasemi *et al.* 2018 [19]	Multimodality Imaging for Precision Oncology	Personalized Medicine	Tailoring treatment strategies to individual patients based on the unique characteristics of their tumors revealed through multimodality imaging.
10	Ni *et al.* 2020 [20]	Nanoparticles for Imaging and Therapy	Theragnostic	Combining diagnostic and therapeutic functions within a single imaging modality.
11	Ma *et al.* 2024 [21]	Image-Guided Liver Interventions	Minimally Invasive Procedures	Guiding minimally invasive procedures with greater precision, reducing complications, and improving patient.
12	Weissman *et al.* 2011 [22]	Multimodality Imaging	Reduced Healthcare Costs	Early detection and accurate diagnosis with multimodality imaging can potentially reduce overall healthcare costs.
13	Smith *et al.* 2021 [23]	Breast Cancer	Treatment Planning	MRI and CT provide complementary data, enhancing detection and treatment planning.
14	F Deharo *et al*. 2023 [24]	Lung Cancer	Theranostics	PET/MRI offers superior diagnostic accuracy over single-modality approaches.
15	Williams *et al.* 2019 [25]	Prostate Cancer	Treatment Planning	Combined PET/CT/MRI improves staging accuracy and treatment outcomes.
16	Brown *et al.* 2018 [26]	Liver Cancer	Diagnosis and Characterization	Ultrasound and MRI together enhance liver lesion characterization.
17	Davis *et al.* 2017 [27]	Colorectal Cancer	Treatment Monitoring	PET/CT is crucial for accurate staging and monitoring of treatment response.
18	Li, *et al.* 2024 [28]	Head and Neck Cancer	Treatment Planning	Combined imaging improves the precision of tumor delineation and treatment planning.
19	Lee *et al.* 2020 [29]	Brain Tumors	Diagnosis and Tumor Characterization	PET/MRI provides better anatomical and metabolic information for brain tumors.
20	Thompson *et al.* 2109 [30]	Ovarian Cancer	Diagnosis and Early Detection	Multimodal imaging enhances early detection and accurate staging of ovarian cancer.
21	Hu *et al.* 2024 [31]	Pancreatic Cancer	Diagnosis and Treatment Monitoring	PET/CT is effective in detecting metastatic lesions and assessing treatment response and also provides AI-based analysis.
22	Kumar *et al.* 2017 [32]	Pediatric Cancer	Early Detection	PET/MRI reduces radiation exposure while providing comprehensive diagnostic information.
23	L White *et al*. 2021 [33]	Radiomics and Lung Cancer	Emerging role in lung cancer research	Imaging biomarkers improve the accuracy of lung cancer diagnosis and radiomics.
24	Anderson *et al.* 2020 [34]	Melanoma	Treatment Monitoring	PET/CT helps in detecting metastasis and monitoring treatment efficacy.
25	A Morshid *et al*. 2021 [35]	Artificial intelligence in cancer imaging	Imaging biomarkers and clinical management	Multimodal imaging improves the characterization and staging through AI cancer imaging.
26	Harris *et al.* 2018 [36]	Thyroid Cancer	Diagnosis and Treatment Monitoring	MRI and PET enhance the detection of recurrent thyroid cancer.
27	Zhang *et al.* 2017 [37]	Bladder Cancer	Diagnosis and Characterization	Multimodal imaging aids in the accurate staging and assessment of bladder tumors.
28	Clark *et al.* 2021 38]	Esophageal Cancer	Early Detection	PET/MRI provides a comprehensive evaluation of tumor invasion and lymph node involvement.
29	Nguyen *et al.* 2020 [39]	Soft Tissue Sarcomas	Diagnosis and Treatment Monitoring	Multimodal imaging assists in surgical planning and assessing treatment response.
30	Lewis *et al.* 2019 [40]	Gynecologic Cancer	Diagnosis and Tumor Characterization	Combined CT and MRI improve the detection and characterization of gynecologic tumors.
31	Perez *et al.* 2018 [41]	Hepatocellular Carcinoma		PET/CT enhances the detection of distant metastases and recurrence.
32	Hao *et al.* 2023 [42]	Bone Cancer	Early Detection	Hybrid imaging provides a comprehensive evaluation of bone tumors and metastases.

**Table 2:** Regional variations in major cancer risk factors.

**S.No.**	**Aspect**	**Details**		**Year and References**
**1.**	Number of Patients	12.7 million cases globally		**2010 (**Global Cancer Observatory**)**
		16.3 million cases globally		2023 (WHO)
**2.**	Death Statistics	Male: 5.7 million deaths		2023 (WHO)
		Female: 4.5 million deaths		
**3.**	Most Affected Areas	United States	Lung, breast, prostate, and colorectal cancers (High incidence rates)	2023, CDC
		China	Liver, stomach, and esophageal cancers (Highest new cases)	2023, NCC, China
		India	Breast, cervical, and oral cancers (Increasing)	2023, ICMR India
		Europe	Lung, breast, and colorectal cancers; smoking and alcohol consumption.	2023, ECIS, UK
**4.**	Main Causes by Area	United States	Tobacco use, obesity, dietary factors, physical inactivity	2023, CDC
		China	Tobacco use, chronic infections (HBV, HCV), dietary habits, environmental pollution	2023, NCC, China
		India	Tobacco, cervical, dietary factors, alcohol consumption.	2023, ICMR India
		Europe	Tobacco, alcohol, dietary factors, obesity, occupational exposures	2023, ECIS, UK

**Table 3:** Cancer care analysis through imaging modalities (year-wise).

**S.No.**	Year	**Imaging Modalities**	**Findings Percentage**	**Availability/ Annotations**
1.	2014	Types of imaging (CT, MRI, PET)	CT (40%), MRI (30%), PET (15%)	55% with PET and advanced PET scanners
2.	2015	Types of imaging (CT, MRI, PET)	CT (39%), MRI (32%), PET (16%)	58% with PET and Improved MRI Resolution
3.	2016	Types of imaging (CT, MRI, PET)	CT (38%), MRI (34%), PET (17%)	60% with PET and Enhanced CT
4.	2017	Types of imaging (CT, MRI, PET)	CT (37%), MRI (36%), PET (18%)	62% with PET, hybrid PET/MRI systems
5.	2018	Types of imaging (CT, MRI, PET)	CT (36%), MRI (38%), PET (20%)	65% with PET, AI-enhanced
6.	2019	Types of imaging (CT, MRI, PET)	CT (35%), MRI (40%), PET (22%)	68% with PET, Advances in MRI
7.	2020	Types of imaging (CT, MRI, PET)	CT (34%), MRI (42%), PET (23%)	70% of facilities with PET, Expansion of AI
8.	2021	Types of imaging (CT, MRI, PET)	CT (33%), MRI (44%), PET (25%)	72% with PET, Enhanced AI
9.	2022	Types of imaging (CT, MRI, PET)	CT (32%), MRI (46%), PET (27%)	75% with PET, portable imaging devices
10.	2023	Types of imaging (CT, MRI, PET)	CT (31%), MRI (48%), PET (30%)	77% with PET, advanced AI, and machine learning

**Table 4:** Comparative analysis of imaging techniques for cancer diagnosis and management.

**S.No.**	**Imaging Modalities**	**Description**	**Comments/** **Limitations**	**Benefits**	**Drawbacks**
**1.**	X-Ray	Uses ionizing radiation to create images of internal structures.	Carries a small radiation dose.	Readily available, inexpensive, and good for detecting fractures	Limited soft tissue contrast and cannot differentiate between different types of tissue.
**2.**	CT Scan	Uses multiple X-ray images to create detailed cross-sectional views of the body.	Not suitable for pregnant women	Best for bones, soft tissues, and blood vessels. Helpful for staging cancer and treatment planning.	More expensive than an X-ray. Carries a higher radiation dose.
**3.**	MRI	Uses strong magnetic fields and radio waves to create detailed images of organs.	Not used for patients with medical implants.	Excellent soft tissue contrast, allowing for better visualization	Expensive and time-consuming procedure. Claustrophobic for some patients.
**4.**	Ultrasound	Uses high-frequency sound waves to create images of organs and soft tissues.	No use of frequently	Real-time imaging, Safe for pregnant women, and frequent monitoring.	Limited penetration depth and is not suitable for deep structure visualizing.
**5.**	PET Scan	Uses radioactive tracers to measure metabolic activity in tissues.	Not always specific to cancer	Can detect tumors based on their increased metabolic activity.	Expensive and requires access to a cyclotron for tracer production.
**6.**	SPECT Scan	Similar to PET scans, it uses radioactive tracers to create 3D images of specific processes.	Not as detailed as PET scans.	Information on blood flow, bone health, and certain cancers.	Less widely available than PET scans. Carries a small radiation dose.

**Table 5:** Cancer care analysis based on different categories.

**S.No.**	**Category**	**Important Findings**	**Observations**
**1.**	**Patient Demographics**	Above 30-85 years of age and 60% female, 40% male, 50% below the poverty line	Varies by cancer type
**2.**	**Cancer Type**	Breast, Lung, Prostate, 30% stage I, 20% stage IV	Prevalence and survival rates differ by type
**3.**	**Outcomes**	1-year 80%, 5-year 50% and 70% report good quality of life	Survival rates improve with advancements
**4.**	**Resource Utilization**	Approx. 500 patent in 4 days and every 3 months for follow-ups and treatment cost is $50,000 per patient	Important for monitoring and managing care
**5.**	**Treatment Modalities**	Surgery (70% of patients)	Effectiveness varies by cancer type
		Radiation Therapy (50% of patients)	Side effects and efficacy vary
		Chemotherapy (6 cycles, 60% response rate)	Impacts quality of life
		Targeted Therapy (30% of patients, specific drugs)	Often tailored to genetic mutations
		Immunotherapy (25% of patients, 40% response)	Emerging treatment, varied effectiveness

**Table 6:** Global statistics WHO cancer care analysis (year-wise).

S.No.	Year	**Category**	**Important Findings**	**Remarks**
1.	2014	Deaths	8.2 million deaths	Baseline data for comparison
		Affected Areas	Global	
		Infected Patients	14.1 million cases	
		Cause	Lung, Breast, Colorectal	
		Resource	60% of facilities with radiotherapy	
2.	2015	Deaths	8.4 million deaths	Cancer affects regions worldwide
		Affected Areas	Global	
		Infected Patients	14.5 million cases	
		Cause	Lung, Breast, Colorectal	
		Resource	62% of facilities with radiotherapy	
3.	2016	Deaths	8.6 million deaths	Resource availability varies by region
		Affected Areas	Global	
		Infected Patients	14.9 million cases	
		Cause	Lung, Breast, Colorectal	
		Resource	65% of facilities with radiotherapy	
4.	2017	Deaths	8.8 million deaths	Cancer affects regions worldwide
		Affected Areas	Global	
		Infected Patients	15.2 million cases	
		Cause	Lung, Breast, Colorectal	
		Resource	67% of facilities with radiotherapy	
5.	2018	Deaths	9.0 million deaths	Resource availability varies by region
		Affected Areas	Global	
		Infected Patients	15.6 million cases	
		Cause	Lung, Breast, Colorectal	
		Resource	70% of facilities with radiotherapy	
6.	2019	Deaths	9.2 million deaths	Cancer affects regions worldwide
		Affected Areas	Global	
		Infected Patients	16.0 million cases	
		Cause	Lung, Breast, Colorectal	
		Resource	72% of facilities with radiotherapy	
7.	2020	Deaths	10.0 million deaths	Impacted by pandemic disruptions
		Affected Areas	Global	
		Infected Patients	19.3 million cases	
		Cause	Lung, Breast, Colorectal	
		Resource	75% of facilities with radiotherapy	
8.	2021	Deaths	10.2 million deaths	Resource availability varies by region
		Affected Areas	Global	
		Infected Patients	19.6 million cases	
		Cause	Lung, Breast, Colorectal	
		Resource	77% of facilities with radiotherapy	
9.	2022	Deaths	10.5 million deaths	Cancer affects regions worldwide
		Affected Areas	Global	
		Infected Patients	20.0 million cases	
		Cause	Lung, Breast, Colorectal	
		Resource	80% of facilities with radiotherapy	
10.	2023	Deaths	10.7 million deaths	Cancer affects regions worldwide
		Affected Areas	Global	
		Infected Patients	20.5 million cases	
		Cause	Lung, Breast, Colorectal	
		Resource	82% of facilities with radiotherapy	

**Table 7:** Cancer care analysis of death statistics by gender and year.

**S.No.**	**Deaths (Millions)**			**Year**	**Remarks**
	**Male Deaths (Millions)**	**Female Deaths (Millions)**	**Total Deaths (Millions)**		
**1.**	4.3	3.3	7.6	2014	Baseline Data
**2.**	4.4	3.4	7.8	2015	Increase Deaths
**3.**	4.5	3.5	8.0	2016	Continuing Rise
**4.**	4.6	3.6	8.2	2017	Increase Deaths Both
**5.**	4.7	3.7	8.4	2018	Incremental Yearly
**6.**	4.9	3.8	8.7	2019	Mortality Rates
**7.**	5.1	4.0	9.1	2020	Impact COVID-19
**8.**	5.3	4.2	9.5	2021	Continued Rise
**9.**	5.5	4.3	9.8	2022	Increasing Deaths
**10.**	5.7	4.5	10.2	2023	Highest Recorded

**Table 8:** Cancer care analysis of number of patients by gender and year.

**S.No.**	Number of Patients **(Millions)**			**Year**	**Remarks**
	**Male** Patients **(Millions)**	**Female** Patients **(Millions)**	**Total** Patients **(Millions)**		
**1.**	6.5	5.0	11.5	2014	Baseline Data
**2.**	6.7	5.2	11.9	2015	Increase Deaths
**3.**	7.0	5.4	12.4	2016	Continuing Rise
**4.**	7.3	5.6	12.9	2017	Increase Deaths Both
**5.**	7.6	5.8	13.4	2018	Incremental Yearly
**6.**	8.0	6.0	14.0	2019	Mortality Rates
**7.**	8.5	6.3	14.8	2020	Impact COVID-19
**8.**	8.8	6.5	15.3	2021	Continued Rise
**9.**	9.2	6.8	16.0	2022	Increasing Deaths
**10.**	9.5	7.0	16.5	2023	Highest Recorded

**Table 9:** Cancer care analysis of main causes by area by year.

**S.No.**	**Year**	**Country**	**Important Findings**	**Causes**
**1.**	2014	United States	Lifestyle-related factors influencing cancer rates	Tobacco, obesity, dietary factors, and physical inactivity
		China	Liver and stomach cancers	Tobacco, chronic infections, and pollution
		India	Cervical and oral cancers	smoking and chewing, infections (HPV), and dietary factors
		Europe	Lung and colorectal cancers	use of tobacco, alcohol consumption, and dietary factors
**2.**	2015	United States	Lifestyle factors	Tobacco, dietary factors, and physical inactivity
		China	Liver and stomach cancers	Tobacco use, chronic infections (HBV, HCV), dietary habits, and pollution
		India	Cervical and oral cancers	Tobacco use (smoking and chewing), infections (HPV), and dietary factors
		Europe	Lung and colorectal cancers (High)	Tobacco use, alcohol consumption, dietary factors, obesity, and occupation
**3.**	2016	United States	Persistent trends in cancer causes	Tobacco use, obesity, dietary factors, and physical inactivity
		China	High rates of liver and stomach cancers	Tobacco use, Chronic infections (HBV, HCV), Dietary habits, and Pollution
		India	Breast and cervical cancers	Tobacco use (smoking and chewing), Infections (HPV), and Dietary factors
		Europe	Lung and colorectal cancers	Tobacco use, Alcohol consumption, Dietary factors, and Obesity, Occupation
**4.**	2017	United States	Continued major role of lifestyle factors	Tobacco use, Obesity, Dietary factors, and Physical inactivity
		China	Liver and stomach cancers	Tobacco use, Chronic infections (HBV, HCV), Dietary habits, and Pollution
		India	Breast and cervical cancers	Tobacco use (smoking and chewing), Infections (HPV), and Dietary factors
		Europe	Lung and colorectal cancers	Tobacco use, Alcohol consumption, Dietary factors, Obesity, and Occupation
**5.**	2018	United States	Persistent trends in cancer causes	Tobacco use, Obesity, Dietary factors, and Physical inactivity
		China	High prevalence of liver and stomach cancers	Tobacco use, Chronic infections (HBV, HCV), Dietary habits, and Pollution
		India	Increasing rates of breast and cervical cancers	Tobacco use (smoking and chewing), Infections (HPV), and Dietary factors
		Europe	Consistent incidence of lung and colorectal cancers	Tobacco use, Alcohol consumption, Dietary factors, Obesity, and Occupation
**6.**	2019	United States	Persistent lifestyle-related causes	Tobacco use, Obesity, Dietary factors, and Physical inactivity
		China	High rates of liver and stomach cancers	Tobacco use, Chronic infections (HBV, HCV), Dietary habits, and Pollution
		India	Rising breast and cervical cancers	Tobacco use (smoking and chewing), Infections (HPV), and Dietary factors
		Europe	High incidence of lung and colorectal cancers	Tobacco use, Alcohol consumption, Dietary factors, Obesity, and Occupation
**7.**	2020	United States	Impact of COVID-19 on cancer care	Tobacco use, Obesity, Dietary factors, and Physical inactivity
		China	Persistent high rates of liver and stomach cancers	Tobacco use, Chronic infections (HBV, HCV), Dietary habits, and Pollution
		India	Increased rates of breast and cervical cancers	Tobacco use (smoking and chewing), Infections (HPV), and Dietary factors
		Europe	Continued high incidence of lung and colorectal cancers	Tobacco use, Alcohol consumption, Dietary factors, Obesity, and Occupation
**8.**	2021	United States	Persistent lifestyle-related causes	Tobacco use, Obesity, Dietary factors, and Physical inactivity
		China	High rates of liver and stomach cancers	Tobacco use, Chronic infections (HBV, HCV), Dietary habits, and Pollution
		India	Rising breast and cervical cancers	Tobacco use (smoking and chewing), Infections (HPV), and Dietary factors
		Europe	High incidence of lung and colorectal cancers	Tobacco use, Alcohol consumption, Dietary factors, Obesity, and Occupation
**9.**	2022	United States	Continued impact of lifestyle factors	Tobacco use, Obesity, Dietary factors, and Physical inactivity
		China	Persistent high rates of liver and stomach cancers	Tobacco use, Chronic infections (HBV, HCV), Dietary habits, and Pollution
		India	Increasing rates of breast and cervical cancers	Tobacco use (smoking and chewing), Infections (HPV), and Dietary factors
		Europe	High and stable incidence of lung and colorectal cancers	Tobacco use, Alcohol consumption, Dietary factors, Obesity, and Occupation
**10.**	2023	United States	Persistent lifestyle-related causes	Tobacco use, Obesity, Dietary factors, and Physical inactivity
		China	High rates of liver and stomach cancers	Tobacco use, Chronic infections (HBV, HCV), Dietary habits, and Pollution
		India	Rising breast and cervical cancers	Tobacco use (smoking and chewing), Infections (HPV), and Dietary factors
		Europe	High incidence of lung and colorectal cancers	Tobacco use, Alcohol consumption, Dietary factors, Obesity, and Occupation

**Table 10:** The effects of radiation on the human body.

**S.No.**	**Effect on Human**	**Radiation Range (mSv)**	**Disorders**
1	Cells repair minor damage effectively.	0–50 mSv	None detectable; possible long-term cancer risk.
2	Low-level exposure may lead to slight blood changes	50–200 mSv	Increased risk of cancer over time.
3	Temporary radiation illness: fatigue, headache; blood cell production may be reduced.	200–1,000 mSv	Increased cancer risk; possible immune effects.
4	Moderate radiation illness: nausea, vomiting, higher blood and immune system damage.	1,000–2,000 mSv	Potential sterility; significant cancer risk.
5	Severe radiation sickness: more intense symptoms, skin damage, and potential central nervous effects.	2,000–6,000 mSv	High mortality risk without treatment; organ failure.
6	Life-threatening exposure: significant organ damage.	6,000–10,000 mSv	Death is likely within weeks without treatment.
7	Fatal exposure: severe damage to all tissues, nervous system failure.	>10,000 mSv	Immediate death or within hours to days.

**Table 11:** Typical radiation dose levels from various medical imaging techniques.

**S.No.**	**Radiation Range (mSv)**	**Imaging Technique**	**Effect on Human**	**Disorders**
**1**	0 mSv (Non-ionizing radiation)	Ultrasound	No ionizing radiation	None detectable.
**2**	0 mSv (Non-ionizing radiation)	MRI	Safe for most patients	None detectable.
**3**	0.1 mSv	X-Ray (Chest X-Ray)	Minimal risk; no immediate effects.	Negligible cancer risk over time.
**4**	0.005–0.01 mSv	X-Ray (Dental)	Extremely low dose; safe for diagnostic purposes.	None detectable.
**5**	0.4 mSv	Mammography	Low risk; safe for breast screening.	Minimal cancer risk over time.
**6**	2–10 mSv (depending on the area)	Computed Tomography	No immediate effects	Slight increase in cancer risk
**7**	10 mSv	CT (Abdomen and Pelvis)	Moderate radiation	Increased risk with repeated
**8**	5–15 mSv	CT (Cardiac or Chest)	Moderate to high exposure	Long-term cancer risk in rare cases.
**9**	10–25 mSv (with CT combination)	Positron Emission Tomography	High exposure; generally safe when medically indicated.	Long-term risk with repeated scans.
**10**	10–50 mSv (per procedure)	Fluoroscopy	Significant exposure, depending on procedure duration.	Increased risk with prolonged use.

## LIST OF ABBREVIATIONS

CCU: Cancer Care Unit
MRI: Magnetic Resonance Imaging
PET: Positron Emission Tomography
CT: Computer Tomography
AI: Artificial Intelligence
NMR: Nuclear Magnetic Resonance
DWI: Diffusion-Weighted Imaging
SPECT: Single-photon emission Computed Tomography
NIR: Near-Infrared
PDT: Photodynamic Therapy
NPs: Nanoparticles
EIT: Electrical Impedance Tomography
WHO: World Health Organization
